# Cohort profile for the STratifying Resilience and Depression Longitudinally (STRADL) study: A depression-focused investigation of Generation Scotland, using detailed clinical, cognitive, and neuroimaging assessments

**DOI:** 10.12688/wellcomeopenres.15538.2

**Published:** 2021-07-16

**Authors:** Tina Habota, Anca-Larisa Sandu, Gordon D. Waiter, Christopher J. McNeil, J. Douglas Steele, Jennifer A. Macfarlane, Heather C. Whalley, Ruth Valentine, Dawn Younie, Nichola Crouch, Emma L. Hawkins, Yoriko Hirose, Liana Romaniuk, Keith Milburn, Gordon Buchan, Tessa Coupar, Mairi Stirling, Baljit Jagpal, Beverly MacLennan, Lucasz Priba, Mathew A. Harris, Jonathan D. Hafferty, Mark J. Adams, Archie I. Campbell, Donald J. MacIntyre, Alison Pattie, Lee Murphy, Rebecca M. Reynolds, Rebecca Elliot, Ian S. Penton-Voak, Marcus R. Munafò, Kathryn L. Evans, Jonathan R. Seckl, Joanna M. Wardlaw, Stephen M. Lawrie, Christopher S. Haley, David J. Porteous, Ian J. Deary, Alison D. Murray, Andrew M. McIntosh

**Affiliations:** 1 University of Aberdeen, Aberdeen, UK; 2 University of Dundee, Dundee, UK; 3 Ninewells Hospital & Medical School, Dundee, UK; 4 University of Edinburgh, Edinburgh, UK; 5 Queen’s Medical Research Institute, Edinburgh, UK; 6 University of Manchester, Manchester, UK; 7 University of Bristol, Bristol, UK

**Keywords:** Cognition, Depression, Generation Scotland, Longitudinal, Neuroimaging, Psychological resilience

## Abstract

STratifying Resilience and Depression Longitudinally (STRADL) is a population-based study built on the Generation Scotland: Scottish Family Health Study (GS:SFHS) resource. The aim of STRADL is to subtype major depressive disorder (MDD) on the basis of its aetiology, using detailed clinical, cognitive, and brain imaging assessments. The GS:SFHS provides an important opportunity to study complex gene-environment interactions, incorporating linkage to existing datasets and inclusion of early-life variables for two longitudinal birth cohorts. Specifically, data collection in STRADL included: socio-economic and lifestyle variables; physical measures; questionnaire data that assesses resilience, early-life adversity, personality, psychological health, and lifetime history of mood disorder; laboratory samples; cognitive tests; and brain magnetic resonance imaging. Some of the questionnaire and cognitive data were first assessed at the GS:SFHS baseline assessment between 2006-2011, thus providing longitudinal measures relevant to the study of depression, psychological resilience, and cognition. In addition, routinely collected historic NHS data and early-life variables are linked to STRADL data, further providing opportunities for longitudinal analysis. Recruitment has been completed and we consented and tested 1,188 participants.

## Introduction

### Why was the study set up?

Major depressive disorder (MDD) affects approximately 13% of the population at least once in their lifetime ^1^, and remains a leading cause of economic burden and non-lethal global disability ^2, 3^ due to its recurrent or chronic nature. At present, MDD diagnosis is based on arbitrary and clinically heterogeneous criteria ^4^. Consequently, and even with optimal management, much of the disability caused by MDD persists ^5^ because of the absence of targeted disease-modifying treatments. The underlying pathophysiology of MDD is believed to be heterogeneous ^6^, with genetic and environmental factors acting to influence disease expression. Thus, it is important for treatment to shift from the current “trial and error” approach, towards precision prevention and stratified medicine based on markedly different disease mechanisms. However, progress in this area has been severely restricted because the aetiology of MDD is complex, and remains poorly understood.

STratifying Resilience and Depression Longitudinally (STRADL) aims to subtype MDD on the basis of its aetiology using detailed clinical, cognitive, and brain imaging assessments. STRADL will examine the interaction between genetic and environmental factors that increase risk and occurrence of different MDD subtypes, and assess common and distinct mechanisms and clinical trajectories of MDD phenotypes. Additionally, STRADL aims to assess individual resilience, or the ability to adapt positively and ‘avoid’ psychopathology despite exposure to known risk factors such as stress, early-life adversity, and family history ^7^. Stratification of MDD will be based on several variables to address its underlying causal and clinical heterogeneity, including: age of onset of MDD; single episode or recurrent depression; obstetric trauma; and developmental factors such as childhood maltreatment, early socioeconomic adversity, and stressful life events. Our key initial predictions are that depression can be stratified on the basis of age of onset into early-onset forms that show a stronger phenotypic and genetic relationship with schizophrenia and other severe mental disorders, and later onsets that show stronger associations with cardiovascular disease and dementia.

STRADL was built on the Generation Scotland: Scottish Family Health Study resource (GS:SFHS) ^8^, which undertook its first major baseline assessments between 2006 and 2011. GS:SFHS is a population-based study of genetic health and complex disease in a cohort of 24,096 individuals, who have been extensively phenotyped for MDD and related traits. This cohort provides an important opportunity to study gene-environment interactions, and remains one of the richest sources of data available, incorporating linkage of existing phenotypic and genomic data, detailed lifestyle and socioeconomic characterisation, extensive eHealth Record linkage ^9^, and the inclusion of two longitudinal birth cohorts – the Walker birth cohort ^10^, and Aberdeen Children of the 1950s (ACONF) ^11^. Table 1 shows data linkages between the current study and existing datasets.

The first wave of STRADL included depression-focused follow-up assessment of GS:SFHS, which involved remote questionnaires that specifically assessed aspects of psychological resilience, coping style, and response to psychological distress; study protocol and cohort characteristics are described elsewhere ^12^. Here, we describe the second wave of STRADL, a depression-focused deep phenotyping face-to-face assessment, using detailed clinical and cognitive tests, and neuroimaging. The results describe the cohort profile and baseline questionnaire and cognitive data, and we provide a summary of key demographic data from the current wave of STRADL, compared to STRADL remote follow-up and wider GS:SFHS baseline assessment. A summary of all data available and the proportion of valid and useable data is also provided. 

**Table 1.** STRADL face-to-face linkage to existing studies and permanently linked datasets.

Database	Description	Data acquired
*GS:SFHS*	GS:SFHS baseline assessment	2006 – 2011
*STRADL*	STRADL remote questionnaire follow-up	2015 – 2017
*Birth cohorts*		
ACONF	Aberdeen Children of the 1950s	1950 – 1956
The Walker Cohort	The Walker Project	1952 – 1966
*Routine linkage*		
Outpatient Attendance (SMR00)	New and follow-up outpatient attendance	1996 – present
General/Acute Inpatient and Day Case (SMR01)	Hospital inpatients discharged from non-obstetric and non- psychiatric specialties	1981 – present
Maternity Inpatient and Day Case (SMR02)	Hospital inpatient discharges from obstetrics specialties	1975 – present
Mental Health Inpatient and Day Case (SMR04)	Psychiatric hospital discharges and diagnostic information	1981 – present
Scottish Cancer Registry (SMR06)	Personal, demographic, and diagnostic information on all new cases of cancer	1958 – present
Neonatal (SMR11)	Neonatal discharges submitted for babies who are sick or have congenital anomalies	1957 – 2002
National Records of Scotland – Deaths	Death registrations	1974 – present
Scottish Drug Misuse Database	Problem drug use	1990 – present
Prescribing Information System	Prescriptions prescribed, dispensed and reimbursed with the community setting	1993 – present

## Methods

### Who is in the cohort?

We aim to study people both with and without depression, and therefore our recruitment targeted the whole GS:SFHS population, not merely people with a depression history. GS:SFHS included participants aged 35–65 years who were identified at random from collaborating medical practices across Scotland, with some family members further afield. Initially, only Glasgow and Tayside areas were involved, but the study was extended in 2010 to include Ayrshire, Arran and Northeast Scotland, with the age range also broadened (to 18–65 years). Participants were included if: they met the age criteria; had capacity to give informed consent; and could identify at least one first-degree relative who would also participate. Follow-up of participants was done through the NHS Scotland Community Health Index (CHI): 7% of the original cohort could not be matched; no participants withdrew; and ~1,200 had died. Those who participated in GS:SFHS were invited to take part in the STRADL remote follow-up based on the following eligibility criteria : they had given consent for re-contact; were living in Scotland; and had a CHI number. 9,618 GS:SFHS responded to the invitation, including 2,460 unrelated individuals and 2,460 families (7,158 individuals) of between 2 and 18 family members.

Participants in the Tayside and Grampian areas who had already taken part in GS:SFHS between 2006–2011, and who were eligible for re-contact, were sent a postal invitation by the University of Dundee Health Informatics Centre (HIC). Included in the invitation was a reply slip to indicate whether the participant would be willing to undergo face-to-face assessment and brain magnetic resonance imaging (MRI), described here. Those who replied positively were contacted by telephone by a researcher at the most local recruitment centre. In Dundee (Tayside) recruitment targeted members of the Walker cohort, and in Aberdeen (Grampian) recruitment initially targeted members of ACONF, due to the rich early-life data already available for these cohorts.

**Figure 1.**
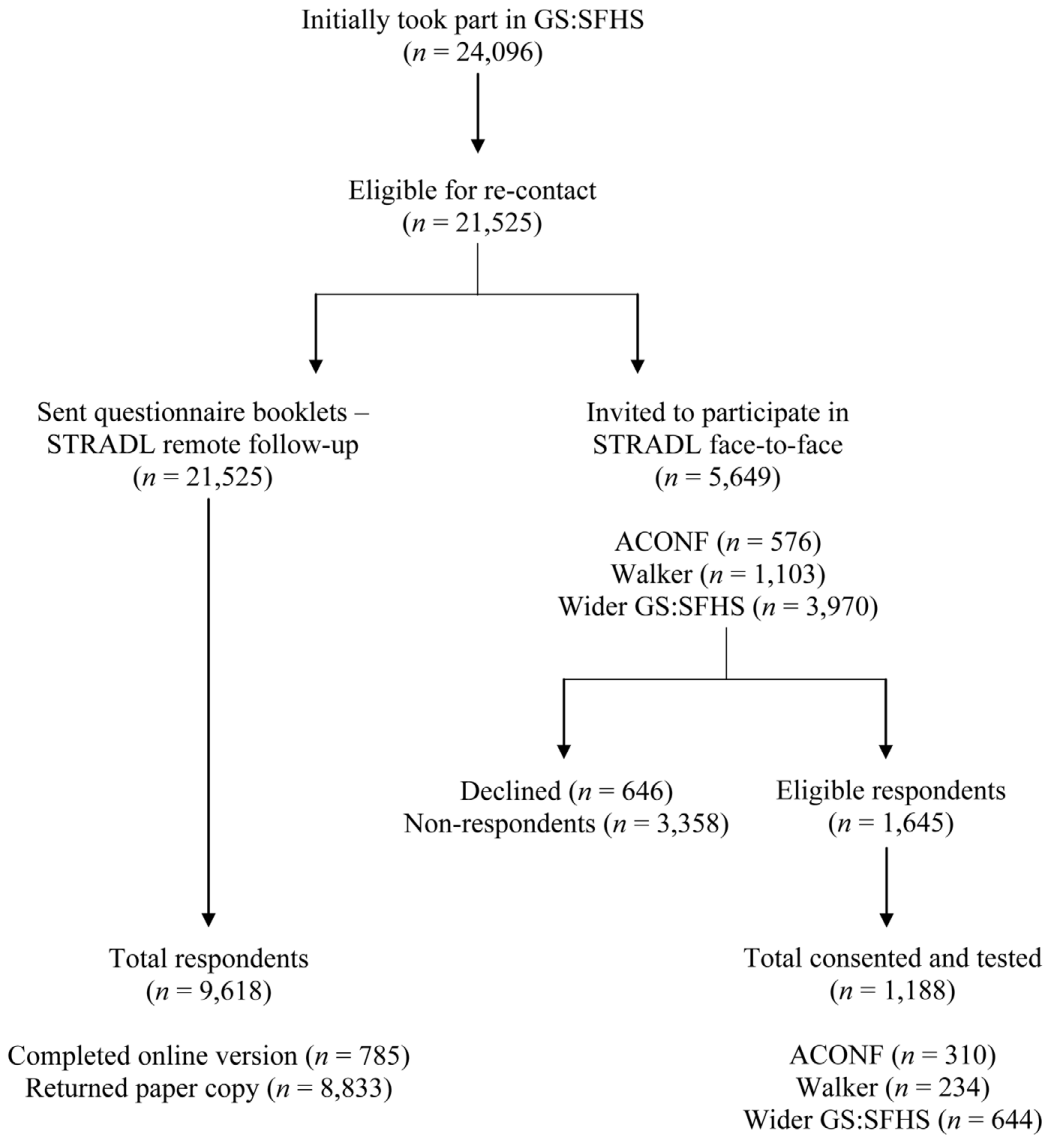
STRADL recruitment flowchart.

In total, 5,649 potential participants were invited to take part in the study; 576 (10.2%) were members of ACONF; 1,103 (19.5%) were members of the Walker cohort; and 3,970 (70.3%) were members of the wider GS:SFHS population. Out of these potential participants, 646 (11.4%) people declined participation at first point of contact with HIC, and we received no reply from 3,358 (59.4%) people, even after sending up to three reminders. Initially, 1,645 (29.1%) people responded positively; however, a further 170 (3.0%) declined once they were contacted by our research team or withdrew before consenting. Recruitment ended in May 2019 and we consented and tested 1,188 (72.2%) of positive respondents across Aberdeen ( *n* = 582) and Dundee ( *n* = 606) sites. This meant that we tested 21% of the *n* = 5,649 who were initially invited to participate. Figure 1 shows the recruitment process and attrition.

### What has been measured and when?

Table 2 shows all variables collected in STRADL face-to-face assessments, and those that were repetitions of the GS:SFHS baseline assessment and STRADL remote questionnaire follow-up. Before any new data were collected, participants signed a consent form permitting data and samples to be shared with other researchers through a secure data management system, and provided permission to be re-contacted in the future for additional research. Consent for linkage of participant data and samples to routine NHS records was previously obtained as part of the original GS:SFHS (05/S1401/89). All subsequent procedures were conducted following an independent, but linked, ethics application (14/SS/0039).

**Table 2.** List of novel and repeated variables collected in STRADL face-to-face assessment, compared to STRADL remote follow-up and GS:SFHS baseline assessment.

Measures	New data in STRADL face-to-face assessment (current)	Repeat from STRADL remote follow-up (2015)	Repeat from GS:SFHS baseline assessment (2006–2011)
*Basic demographics*		✓	✓
*Health and lifestyle*			
Alcohol history		✓	✓
Smoking history		✓	✓
Fatigue	✓		
Loneliness	✓		
Medical and mental health history		✓	✓
List of medications			✓
Current infection(s)	✓		
Cardiovascular health		✓	✓
*Physical measures*			
Height			✓
Weight			✓
Blood pressure			✓
Grip strength	✓		
*Laboratory samples*			
Full Blood Count	✓		
C-Reactive Protein	✓		
Blood – DNA			✓
Saliva – DNA			✓
RNA	✓		
Plasma biomarkers (EDTA)			✓
Plasma biomarkers (LiH)			✓
Hair sample	✓		
*Psychiatric assessment &* *questionnaire data*			
Structured Clinical Interview for DSM Disorders			✓
Brief Resilience Scale		✓	
Cannabis use	✓		
Hospital Anxiety and Depression Scale	✓		
Mood Disorder Questionnaire			✓
Quick Inventory of Depressive Symptomology	✓		
General Health Questionnaire		✓	✓
Eysenck Personality Questionnaire – Revised			✓
International Personality Item Pool	✓		
General Causality Orientations Scale	✓		
Childhood Trauma Questionnaire	✓		
*Cognitive tests*			
Logical Memory test (immediate and delayed)			✓
Digit Symbol Coding			✓
Verbal fluency			✓
Mill Hill Vocabulary test			✓
Matrix Reasoning test	✓		
Bristol Emotion Recognition test	✓		
Affective Go/No-Go task	✓		
Cambridge Gambling task	✓		
*Brain MRI*			
T1-weighted	✓		
NimStim fearful faces task	✓		
Reward task	✓		
Resting state	✓		
Fluid attenuation inversion recover (FLAIR)	✓		
Diffusion tensor imaging (DTI)	✓		
T2-weighted	✓		
Susceptibility weighted imaging (SWI)	✓		

Abbreviations: DSM, Diagnostic and Statistical Manual of Mental Disorders; DNA, Deoxyribonucleic acid; EDTA, Ethylenediaminetetraacetic acid; LiH, Lithium Heparin; RNA, Ribonucleic acid.

At each site participants attended three testing ‘stations’, which involved i) collection of clinical and questionnaire data, and biological samples ii) cognitive assessment, and iii) neuroimaging, the order of which varied at random between participants. Data from the clinical station were collected without a set order; however, cognitive tests were administered in the same order for each participant, and the MRI sequences also remained the same – except for one fMRI task, which was counterbalanced (details described in section *Brain magnetic resonance imaging*). All measures were administered in accordance with rigorous standard operating procedures based on best practice.

### Clinical assessment

Medical history was updated from previous GS:SFHS baseline assessment and any new diagnoses or medical episodes were recorded. General health and lifestyle data were also collected, as were the physical measurements of height, weight, two automated measures of blood pressure, and left- and right-hand grip strength (using a Patterson Medical Jamar hand dynamometer). We collected laboratory blood samples for genetic and additional genomic analyses, including the study of DNA methylation, transcription (RNA) and protein expression. Additionally, detailed questionnaire data were collected that will be used to test the structure of depressive symptoms and their association with each measure.

***Laboratory samples.*** A small sample of hair was collected from the posterior vertex region for longitudinal cumulative cortisol. Cortisol assays from hair samples provide a more stable marker of chronic cortisol exposure compared to cross-sectional blood or urine samples, which show considerable diurnal variation ^13^. Other available assays include cortisone, testosterone, progesterone, and dehydroepiandrosterone. Venepuncture was carried out using a butterfly needle kit. Blood was extracted into the following vacutainers types (analyses in parentheses): 1) EDTA (Full blood count; FBC); 2) clot activator gel for serum separation (C-reactive protein CRP); 3) EDTA (DNA extraction); 4) 2 x Tempus RNA (RNA extraction); 5) EDTA (plasma biomarkers); 6) Lithium Heparin (plasma biomarkers). FBC and CRP samples were taken and sent to NHS laboratories for screening of clinically significant markers of anaemia and inflammation. When blood samples could not be collected, a saliva DNA collection kit (Oragene or GeneFiX) was used instead. These laboratory samples were temporarily stored at each collection site. RNA and blood DNA samples were stored at -80°C, and all others at -20°C, before being sent to the Edinburgh Clinical Research Facility at the University of Edinburgh for analysis and long-term storage. A summary of the completeness of these clinical data is shown in Table 3 and Table 4. FBC and CRP analysis are complete, and other blood and hair samples are in the process of being analysed.

**Table 3.** Summary of demographic and background data, and proportion valid data (n = 1,188).

Measurement		%
*Demographics*	Age	100
	Gender	100
	Highest education attained	99.9
	Occupation	99.8
	Father’s occupation	98.8
	Marital status	99.9
*Health and* *lifestyle*	Alcohol history	99.7
	Smoking history	99.7
	Fatigue (preceding 3 months)	86.7
	Loneliness (preceding 1 week)	99.9
	Medical or mental health history	99.9
	List of medications	100
	Current infection(s)	96.6
*Cardiovascular* *health*	Stroke or transient ischaemic attack	99.9
	Myocardial infarction or angina	99.9
	Other heart disease	99.9
	Peripheral arterial disease	99.9
	Jaw claudication	96.5
	Diabetes	99.9
	Hypertension	100
	Hypercholesterolaemia	99.9

**Table 4.** Summary of deep phenotype data available, and proportion valid data (n = 1,188).

Measurement		%
*Physical measures*	Height	99.9
	Weight	99.9
	Blood pressure – first recording	99.7
	Blood pressure – second recording	97.1
	Grip strength	98.3
*Laboratory samples*	Full Blood Count	96.5
	C-Reactive Protein	97.1
	DNA	99.2
	RNA	96.0
	Plasma biomarkers (EDTA)	97.0
	Plasma biomarkers (LiH)	96.6
	Hair sample	91.0
*Psychiatric assessment*	Structured Clinical Interview for DSM Disorders	100
*Questionnaire data*	Brief Resilience Scale	100
	Cannabis use	99.9
	Hospital Anxiety and Depression Scale	100
	Mood Disorder Questionnaire	100
	Quick Inventory of Depressive Symptomology	99.9
	General Health Questionnaire	99.9
	Eysenck Personality Questionnaire – Revised	99.9
	International Personality Item Pool	100
	General Causality Orientations Scale	98.5
	Childhood Trauma Questionnaire	100
*Cognitive tests*	Logical Memory test (immediate and delayed)	99.9
	Digit Symbol Coding	99.2
	Verbal fluency	99.9
	Mill Hill Vocabulary test	99.9
	Matrix Reasoning test	99.9
	Bristol Emotion Recognition test	98.0
	Affective Go/No-Go task	97.7
	Cambridge Gambling task	98.6
*Brain MRI data*	T1-weighted	91.2
	NimStim fearful faces task	89.1
	Reward task	89.0
	Resting state	90.2
	Fluid attenuation inversion recover (FLAIR)	90.6
	Diffusion tensor imaging (DTI)	89.3
	T2-weighted	89.4
	Susceptibility weighted imaging (SWI)	90.6

*Note*. 96.5% of DNA was extracted via blood sample and 2.7% via saliva sampling. Abbreviations: DSM, Diagnostic and Statistical Manual of Mental Disorders; DNA, Deoxyribonucleic acid; EDTA, Ethylenediaminetetraacetic acid; LiH, Lithium Heparin; RNA, Ribonucleic acid.

***Clinical interview and questionnaire data.*** All participants were assessed for a lifetime history of MDD. We used a research version of the Structured Clinical Interview for DSM-IV disorders (SCID) ^14^ to assess symptoms of mood disorder (including MDD and episodes of mania and hypomania), repeating the GS:SFHS baseline assessment. Diagnostic criteria were based on the Diagnostic and Statistical Manual of Mental Disorders (DSM-IV-TR). For participants who met full criteria for MDD, we assessed if any episode had a post-partum onset, and if criteria for melancholic or atypical MDD subtypes were met. The research version of the SCID was designed to allow assessors to systematically evaluate individuals against the key DSM-IV-TR criteria for unipolar depression and bipolar disorder. The SCID has good reliability, and it is considered the “gold standard” in determining clinical diagnoses and their accuracy ^15^.

Participants also completed a series of short questionnaires that assessed resilience, psychological well-being and mild psychiatric problems, and personality, some of which were repeated after first being completed for GS:SFHS (see Table 2). The Brief Resilience Sca *le* (BRS) ^16^ is a six-item questionnaire used as a measure of psychological resilience, or the ability to ‘bounce back’ from stress. Participants were assessed for a life history of cannabis use using the Drug Use questionnaire developed for UK Biobank ^17^. Those who used cannabis more than once were asked follow-up questions about the frequency and functional impact of their use. Three mood questionnaires were administered: the Mood Disorder Questionnaire (MDQ) ^18^, which is a sensitive screen for bipolar spectrum disorders; the Quick Inventory of Depressive Symptomology (QIDS) ^19^, which is a 16-item inventory designed to assess the severity of depressive symptoms; and the Hospital Anxiety and Depression Scale (HADS) ^20^ anxiety subscale (seven items) was used to screen for symptoms of anxiety. In addition, the General Health Questionnaire (GHQ) ^21^, a 28-item test, was used to assess general psychological well-being on four scales: somatic symptoms; anxiety; social dysfunction; and depression. We used a Likert scoring system for the GHQ to calculate scores for each scale separately, as well as a total score.

We administered two measures that assess core personality traits: we used the neuroticism and extraversion scales from the Eysenck Personality Questionnaire – Revised Short Form (EPQ-R) ^22^, each of which has 12 items; and the International Personality Item Pool (IPIP), Five-Factor Personality Inventory ^23^, which is a 50-item questionnaire that assesses the following core personality traits: extraversion; agreeableness; conscientiousness; emotional stability (the reverse of neuroticism); and imagination/intellect (similar to openness). Additionally, the General Causality Orientations Scale ^24^, which consists of 12 vignettes describing scenarios to determine each person’s orientation of causality ^25^, was used to assess one’s inclination towards being motivated autonomously, externally, or passively.

Finally, we assessed early-life adversity (childhood or adolescent abuse or neglect) using the Childhood Trauma Questionnaire (CTQ) ^26^. This is a 28-item retrospective inventory that assesses three areas of abuse (emotional, physical, and sexual) and two areas of neglect (emotional and physical). The CTQ also includes a minimisation/denial scale that identifies potential underreporting of maltreatment. A mean score was calculated for each measure by totalling the item responses, with appropriate reverse scoring (e.g., GHQ, BRS). Higher scores represent higher levels of psychological distress, personality trait, or childhood trauma, except for the BRS where higher scores indicate greater resilience. Scoring and interpretation of data were based on the administration manual of each test.

### Cognitive testing

The cognitive tests that were applied will be used to assess the cognitive phenotype of depression, and whether genetic risk variants are related to impairment in specific cognitive domains. As with the questionnaire data, some cognitive tests were also repetitions of the GS:SFHS baseline assessment ( Table 2). We included “cold” (emotion-independent) and “hot” (emotion-laden) cognitive tests, given growing evidence for distinct and interacting relationships between depression and measures of hot and cold cognition ^27^.

The cold cognitive test battery included validated and widely used cognitive tests that measure crystallised- and fluid-type cognitive tasks. The Mill Hill Vocabulary test ^28^ was used as a measure of acquired verbal intelligence, and is an estimate of ‘crystallised intelligence’ and peak cognitive ability. The Controlled Oral Word Association task ^29^ was used as a measure of phonemic verbal fluency using three letters (C, F, and L). The Digit Symbol Coding subtest from the Wechsler Adult Intelligence Scale-III ^30^ was used to measure information processing speed. A United Kingdom version of the Logical Memory subtest from the Wechsler Memory Scale-III ^31^ was used to assess verbal memory and provided a measure of immediate and delayed verbal declarative recall. Total scores were created for each cognitive test by adding the number of correct responses; higher scores indicate better performance. The Matrix Reasoning test, a paper adaptation of the computerised version from the COGNITO psychometric examination ^32^, was used to measure perceptual organisation and visuospatial logic. A summary of all mood, personality, and cognitive data and their completeness is shown in Table 4.

Three ‘hot’ cognitive measures were administered on a touchscreen laptop computer. The first task – the Bristol Emotion Recognition Test – consisted of 96 trials (16 of each emotion) that assessed recognition of six basic human facial emotions (happiness, anger, sadness, disgust, surprise, and fear), and biases in the attribution of emotion. The Affective Go/No-Go task comprised 120 trials that assessed behavioural inhibition using facial emotional stimuli (happy, sad, and neutral expressions). Finally, given evidence for impairments in reward processing in depression ^33^, we also included a modified version of the Cambridge Gambling Task, which assesses decision-making, risk-taking behaviour, and reward processing (30 trials). These three tests are described in detail elsewhere ^34, 35^.

### Brain magnetic resonance imaging

The neuroimaging protocol will allow analysis of potential risk factor relationships with brain structure and function, and test neurobiological mechanisms that are associated with depressive symptoms and resilience. In Aberdeen, participants were imaged on a 3T Philips Achieva TX-series MRI system (Philips Healthcare, Best, Netherlands) with a 32 channel phased-array head coil and a back facing mirror (software version 5.1.7; gradients with maximum amplitude 80 mT/m and maximum slew rate 100 T/m/s). A projector and “Presentation” (Neurobehavioural Systems Inc, Berkeley, CA, USA) version 18.1 were used for the presentation of task-based fMRI. In Dundee, participants were scanned using a Siemens 3T Prisma-FIT (Siemens, Erlangen, Germany) with 20 channel head and neck phased array coil and a back facing mirror (Syngo E11, gradient with max amplitude 80 mT/m and maximum slew rate 200 T/m/s). A magnetic resonance compatible LCD screen was used to display fMRI (NordicNeuroLab, Bergen, Norway) task stimuli using “Presentation” version 20.0.

Both centres used the same protocol including structural and functional sequences. The structural sequences collected were as follows: 3D T1-weighted fast gradient echo with magnetisation preparation; 3D T2-weighted fast spin echo; 3D Fluid Attenuation Inversion Recovery (FLAIR); Diffusion Tensor Imaging (DTI); and Susceptibility Weighted Imaging (SWI) or T2*-weighted gradient echo. The functional sequences comprised of two task-based fMRI tasks and a resting state fMRI sequence. The sequence parameters, as well as the order of acquisitions, are presented in Table 5.

**Table 5.** Magnetic resonance imaging (MRI) sequences and their characteristics acquired in Aberdeen and Dundee.

MR sequences ^a^	Aberdeen	Dundee
**3D T1-weighted** (1)	160 sagittal slices TR = 8.2 ms * TE = 3.8 ms TI = 1031 ms FA = 8° FOV = 240 mm matrix size = 240 × 240 voxel size = 1.0 × 1.0 × 1.0 mm ^3^ acquisition time = 5 min 38 s	208 sagittal slices TR = 1740 ms TE = 2.62 ms TI = 900 ms FA = 8° FOV = 256 mm matrix size = 256 × 256 voxel size = 1.0 × 1.0 × 1.0 mm ^3^ acquisition time = 4 min 3 s
**3D T2-weighted** (8)	360 sagittal slices TR = 2500 ms TE = 314 ms FA = 90° FOV= 250 mm matrix size = 252 × 250 voxel size = 0.5 × 0.5 × 0.5 mm ^3^ acquisition time = 7 min 17 s	320 sagittal slices TR = 3200 ms TE = 408 ms FA = variable FOV = 256 mm matrix size = 256 × 256 voxel size = 0.5 × 0.5 × 0.5 mm ^3^ acquisition time = 7 min 10 s
**3D FLAIR** (5)	160 sagittal slices TR = 8000 ms TE = 349 ms TI = 2400 ms FOV = 240 mm matrix size = 240 × 238 voxel size 0.94 × 0.94 × 1.00 mm ^3^ acquisition time = 4 min 32 s	160 sagittal slices TR = 5000 ms TE = 386 ms TI = 1800 ms FOV = 256 mm matrix size = 256 × 256 voxel size 1.00 × 1.00 × 1.00 mm ^3^ acquisition time = 4 min 37 s
**DTI** (7)	60 axial slices TR = 7010 ms TE = 90 ms FA = 90° FOV = 220 mm matrix size = 96 × 94 voxel size = 2.3 × 2.3 × 2.3 mm ^3^ 64 non-collinear gradient directions (b = 1200 s/mm2) eight unweighted (b = 0) acquisition time = 9 min 28s	60 axial slices TR = 7100 ms TE = 87 ms FA = 90° FOV = 220 mm matrix size = 96 × 94 voxel size = 2.3 × 2.3 × 2.3 mm ^3^ 64 non-collinear gradient directions (b = 1200 s/mm2) eight unweighted (b = 0) acquisition time = 8 min 54 s
T2 * weighted or SWI (6)	130 axial slices TR = 31 TE = 7.2/13.4/19.6/25.8 ms FA = 17° FOV = 230 mm matrix size = 384 × 316 voxel size = 0.3 × 0.3 × 1 mm ^3^ acquisition time = 4 min 29 s	144 axial slices TR = 28 ms TE = 20 ms FA = 17° FOV = 230 mm matrix size = 320 × 160 voxel size = 0.4 × 0.4 × 1 mm ^3^ acquisition time = 5 min 17 s
**fMRI sequences** (2, 3, 4)	32 axial slices TR = 1560 ms TE = 26 ms FA = 70° FOV = 217 mm matrix size = 64 × 64 voxel size = 3.4 × 3.4 × 4.5 mm ^3^ total acquisition time = 24 min 13 s	32 axial slices TR = 1560 ms TE = 22 ms FA = 70° FOV = 217 mm matrix size = 64 × 64 voxel size = 3.4 × 3.4 × 4.5 mm ^3^ total acquisition time = 24 min 13 s

^a^Order of acquisition is presented in parentheses. *8.2 ms × 240 phase encoding steps = 1968 ms. Abbreviations: TR, repetition time; TE, echo time; TI, inversion time; FA, flip angle; FOV, field of view; DTI, diffusion weighted tensor images; FLAIR, fluid attenuation inversion recovery; SWI, susceptibility weighted imaging.

T1-weighted images of the brain were used to assess brain regional volumes, cortical thickness, gyrification index, voxel-based morphometry analysis, certain lesions such as lacunes, cortical and larger subcortical infarcts, and will also serve as the basis for co-registration with other sequences. A 3D T2-weighted sequence was used to detect lacunes, perivascular spaces, cortical and subcortical infarcts, and other morphological measurements, such as hippocampal subfield extraction. A 3D FLAIR was used to detect white matter hyperintensities. SWI data, for the determination of brain microbleeds, basal ganglia mineralisation, and cortical superficial siderosis, were acquired using a 3D multi-echo gradient-echo sequence in Aberdeen and a single-echo protocol in Dundee. Phase and magnitude data were saved for the calculation of T2* relaxation. All vascular lesions listed above are defined in the Standards for Reporting Vascular changes on Neuroimaging standards ^36^. All structural images were reviewed by a neuroradiologist for visual analysis of vascular changes and incidental findings. Whole-brain DTI were recorded to allow assessment of microstructural integrity of white matter including fibre direction and structural connectivity. This protocol reflects established neuroimaging approaches as used in several large cohort studies of ageing and of cerebrovascular diseases ^37, 38^.

There were two task-based fMRI sequences: an implicit emotional processing task (fearful versus neutral faces), and a modified version of an instrumental reward task with an additional choice value component. Both of these, as well as resting state fMRI, were acquired at 30 degrees away from the anterior commisure–posterior commisure (AC-PC), towards the coronal plane. The fearful faces from NimStim ^39^ facial stimuli set assessed emotional-limbic circuitry through a block fMRI design, and measures the brain’s neural responses to the viewing of fearful faces in the absence of learning. In order to avoid a gender bias of the images, two versions of the tasks were used, counterbalanced across participants. The Reward task measured reward-related brain activity using an event-related fMRI design in a reinforcement learning context. The resting state fMRI was used to investigate functional connectivity and brain networks.

## Results

### What are the key findings?

Here, we report findings for the complete data set including 1,188 participants. Table 6 shows some demographic similarities and differences between the current STRADL cohort and existing samples. More specifically, the median age of the STRADL sample was 62 years, which is older compared to both STRADL remote follow-up and wider GS:SFHS populations. Analysis of group differences for age showed that participants who are part of ACONF were generally older ( *M* = 62.32; *SD* = 1.55) compared to the Walker cohort and wider GS:SFHS participants ( *ts* ≥ 6.28; ps < .001). However, age did not differ ( *t* = 1.83; *p* = .068) between participants in the Walker cohort ( *M* = 59.48; *SD* = 6.76) and those in GS:SFHS ( *M* = 58.28; *SD* = 12.63). Gender distributions were comparable to existing data, with 59% being female, and our sample had higher levels of education (university-level education = 40%), compared to existing data. Furthermore, based on SCID interviews, a higher proportion of STRADL participants were diagnosed with a lifetime history of mood disorder (30.7%), compared to GS:SFHS (13.2%). Out of the total sample, 28.8% received a diagnosis of MDD, and a further 1.9% of bipolar disorder. Recurrent mood disorder was present in 72.7%, and melancholic features (56%) were dominant in the group. Of those with a diagnosis, 71% were female. Overall, however, the cohort was of good psychological health at the time of assessment, as indicated by mean scores on the GHQ, HADS, and QIDS ( Table 7), which fell below the thresholds for the presence of psychological distress. Cognitive scores across all measures were normally distributed. Psychological health, personality, and cognitive scores are presented in Table 7.

**Table 6.** Comparison of demographic data between STRADL face-to-face assessment, STRADL remote follow-up, and wider GS:SFHS baseline assessment.

Variable	STRADL face-to-face assessment ( *n* = 1,188)	STRADL remote follow-up ^12^ ( *n* = 9,618)	GS:SFHS baseline assessment ^8^ ( *n* = 21,525)
Median age (years)			
Male	62	54	47
Female	61	52	48
Gender (%) (female)	59	62	59
Employment (%) (those aged up to 75 years)			
Unemployed	3	4	2
Retired	32	18	15
Employed	65	71	63
Education (%)			
University-level degree	40	37	33
No qualification	25	7	5

*Note*. Data were extracted from time of each assessment point.

**Table 7.** Baseline questionnaire and cognitive measures (n = 1,188).

Variable	Maximum score	Mean ( *SD*)	Range
***Questionnaire data***			
BRS	30	21.46 (4.89)	0–30
HADS – anxiety	21	4.15 (3.52)	0–20
QIDS – total	27	4.64 (3.65)	0–22
General Health Questionnaire			
GHQ – somatic symptoms	21	3.95 (3.23)	0–21
GHQ – anxiety	21	3.24 (3.32)	0–21
GHQ – social dysfunction	21	7.18 (2.01)	0–21
GHQ – depression	21	0.81 (2.39)	0–21
GHQ – total	84	15.15 (8.56)	2–75
EPQ-R – neuroticism	12	3.40 (3.19)	0–12
EPQ-R – extraversion	12	7.05 (3.80)	0–12
International Personality Item Pool			
IPIP – extraversion	50	31.17 (7.45)	11–50
IPIP – agreeableness	50	41.50 (5.34)	20–50
IPIP – conscientiousness	50	37.62 (6.04)	11–50
IPIP – emotional stability	50	33.58 (7.85)	10–50
IPIP – intellect	50	34.78 (5.58)	11–50
Childhood Trauma Questionnaire			
CTQ – physical abuse	25	6.11 (2.41)	0–25
CTQ – sexual abuse	25	6.04 (3.57)	0–25
CTQ – emotional abuse	25	7.00 (3.47)	0–25
CTQ – physical neglect	25	6.35 (2.36)	0–21
CTQ – emotional neglect	25	8.56 (4.21)	0–25
***Cognitive data***			
Logical Memory test – immediate	25	16.34 (3.63)	4–24
Logical Memory test – delayed	25	15.40 (3.91)	3–24
Logical Memory test – combined	50	31.74 (7.29)	9–48
Digit Symbol Coding	133	68.15 (15.24)	24–116
Verbal fluency test – combined	-	42.98 (12.02)	12–88
Mill Hill Vocabulary test	44	31.56 (4.09)	16–44
Matrix Reasoning test	15	8.26 (2.39)	1–15

Abbreviations: BRS, Brief Resilience Scale; CTQ, Childhood Trauma Questionnaire; EPQ-R, Eysenck Personality Questionnaire – Revised; GHQ, General Health Questionnaire; HADS, Hospital Anxiety and Depression Scale; IPIP, International Personality Item Pool; QIDS, Quick Inventory of Depressive Symptomology.

## Discussion

### Strengths and limitations

STRADL data have been robustly collected on a wide range of key phenotypes that allow epidemiological study of depression and resilience in a population-based cohort. The MRI and detailed depression phenotyping protocol described here was cross-sectional; however, STRADL can provide longitudinal measures of cognition, personality, and psychological health. This is because many of the cognitive tests applied in STRADL are deliberately the same as those used at the GS:SFHS baseline assessment, as well as some personality and mood measures, as shown in Table 2. The availability of repeated cognitive and questionnaire testing allows us to assess potential determinants of change in cognition and psychological health. Similarly, routine NHS data, and ACONF and Walker cohorts’ early-life variables, are linked to STRADL data, providing further opportunities for longitudinal predictors on depression and resilience across the full life-course. However, a limitation of these data is that, as the present study is based on ages of different cohorts including people born in the 1950s onwards, early life variables are likely to have been influenced by cultural and societal norms of that time. For example, people born in the 1950s and 1960s may not have considered housing circumstances such as crowdedness as an ‘adverse experience’, contrary to more contemporary perspectives. These possible confounding variables will be considered in downstream analyses, but it may be difficult to completely mitigate their effects.

A further limitation of this study includes possible selection bias as we tested only 21% of the total pool of eligible and invited participants ( *n* = 5,649). As with many longitudinal population studies, participants in this cohort were more likely to be of good health, and come from more advantaged backgrounds such as higher education and better socioeconomic circumstances than the population in general – findings that are similar to GS:SFHS and STRADL remote follow-up cohort profiles ^8, 12^, and UK Biobank ^17^. Further, because our cohort was of good psychological health at the time of testing, it seems possible that we did not capture as many people with poorer psychological health as what might be available in the wider cohort. Notably, however, participants from a range of health and demographic backgrounds were represented in this group.

### Ethical approval and consent

Ethical approval for the study was obtained from the Scotland A Research Ethics Committee (REC reference number 14/55/0039) and the local Research and Development offices. All participants provided written informed consent prior to the collection of any data or samples.

## Data availability

All data underlying the results are available as part of the article and no additional source data are required.

A phenotype data dictionary is available and open access genome-wide association study summary statistics can be downloaded. Non-identifiable information from the GS:SFHS cohort is available to researchers in the UK and to international collaborators through application to the Generation Scotland Access Committee ( access@generationscotland.org) and through the Edinburgh Data Vault ( https://doi.org/10.7488/8f68f1ae-0329-4b73-b189-c7288ea844d7). Generation Scotland operates a managed data access process including an online application form, and proposals are reviewed by the Generation Scotland Access Committee. The data and samples collected by the STRADL study have been incorporated in the main Generation Scotland dataset and governance process. Summary information to help researchers assess the feasibility and statistical power of a proposed project is available on request by contacting resources@generationscotland.org.
