# Supplementary material for: Cohort profile for the STratifying Resilience and Depression Longitudinally (STRADL) study: A depression-focused investigation of Generation Scotland, using detailed clinical, cognitive, and neuroimaging assessments
Source: Wellcome Open Res. 2021 Jul 16;4:185. Originally published 2019 Nov 25. [Version 2] doi: 10.12688/wellcomeopenres.15538.2 (PMC8857525; doi:10.12688/wellcomeopenres.15538.2)
Supplement: Peer review history

# Peer review: Reviewer response for version 2

**DOI:** 10.21956/wellcomeopenres.18737.r45009

**Published:** 2021-07-23

**Authors:** Debbie A. Lawlor

**Affiliations:** 1 Medical Research Council Integrative Epidemiology Unit, Population Health Sciences, Bristol Medical School, University of Bristol, Bristol, UK

The authors have responded to my comments and I consider this a useful cohort profile

Is the work clearly and accurately presented and does it cite the current literature?

Partly

If applicable, is the statistical analysis and its interpretation appropriate?

Not applicable

Are all the source data underlying the results available to ensure full reproducibility?

Yes

Is the study design appropriate and is the work technically sound?

Partly

Are the conclusions drawn adequately supported by the results?

Partly

Are sufficient details of methods and analysis provided to allow replication by others?

Partly

Reviewer Expertise:

Epidemiology

I confirm that I have read this submission and believe that I have an appropriate level of expertise to confirm that it is of an acceptable scientific standard.

# Peer review: Reviewer response for version 1

**DOI:** 10.21956/wellcomeopenres.17011.r38261

**Published:** 2020-04-07

**Authors:** Helen Lavretsky

**Affiliations:** 1 Department of Psychiatry, University of California, Los Angeles (UCLA), Los Angeles, CA, USA

The reported study, STratifying Resilience and Depression Longitudinally (STRADL) is a population-based study built on the Generation Scotland: Scottish Family Health Study (GS:SFHS) resource. The aim of STRADL is to subtype major depressive disorder (MDD) on the basis of its etiology, using detailed clinical, cognitive, and brain imaging assessments. Recruitment has been completed and we consented and tested 1,188 participants.

 The manuscript describes the methods of data collection in STRADL included: socio-economic and lifestyle variables; physical measures; questionnaire data that assesses resilience, early-life adversity, personality, psychological health, and lifetime history of mood disorder; laboratory samples; cognitive tests; and brain magnetic resonance imaging. Some of the questionnaire and cognitive data were first assessed at the GS:SFHS baseline assessment between 2006-2011, thus providing longitudinal measures of depression and resilience. The presented results are purely description of the sample. Overall, the study has a lot of potential in providing links to the biomarkers and predictors of depression and resilience, including personality traits, early trauma exposure, and genetic vulnerability. However, it would help to provide clarification about study initial hypotheses and the analytical plan.

 The title of the study implies that stratification can be made between depression and resilience longitudinally. Nothing in the description explains what will be done about subtyping MDD (only unipolar and bipolar depression diagnoses are mentioned), or what would it take to stratify by resilience and depression.

Is the work clearly and accurately presented and does it cite the current literature?

Yes

If applicable, is the statistical analysis and its interpretation appropriate?

Yes

Are all the source data underlying the results available to ensure full reproducibility?

Yes

Is the study design appropriate and is the work technically sound?

Yes

Are the conclusions drawn adequately supported by the results?

No

Are sufficient details of methods and analysis provided to allow replication by others?

Yes

Reviewer Expertise:

Resillience, late life depression, biomarkers

I confirm that I have read this submission and believe that I have an appropriate level of expertise to confirm that it is of an acceptable scientific standard.

# Response

**Published:** 2021-06-08

**Authors:** Tina Habota

**Affiliations:** University of Aberdeen, UK

*Response:*

 Thank you for your comments above. We have amended our manuscript to include further clarification about the study hypotheses and focus of stratification of MDD under the section "Why was the study set up?":

*“* *Stratification of MDD* *will be based on several variables to address its underlying causal and clinical heterogeneity, including: age of onset of MDD; single episode or recurrent depression; obstetric trauma; and developmental factors such as childhood maltreatment, early socioeconomic adversity, and stressful life events.* *Our key initial predictions are that depression can be stratified on the basis of age of onset into early-onset forms that show a stronger phenotypic and genetic relationship with schizophrenia and other severe mental disorders, and later onsets that show stronger associations with cardiovascular disease and dementia* *”.*

# Peer review: Reviewer response for version 1

**DOI:** 10.21956/wellcomeopenres.17011.r37159

**Published:** 2020-02-24

**Authors:** Debbie A. Lawlor

**Affiliations:** 1 Medical Research Council Integrative Epidemiology Unit, Population Health Sciences, Bristol Medical School, University of Bristol, Bristol, UK

This profile provides details of the second (face-to-face) assessment of the STRADL (STratifying Resilience and Depression Longitudinally) study. STRADL is itself a sub-study of the Generation Scotland – Scottish Family Health Study (GS-SFHS). The GS-SFHS is a truly impressive resource for the scientific community and this profile is a very valuable paper updating the community on some detailed and novel assessments within a subgroup of that resource.

 My main comments are related to trying to improve the value to the wider scientific community and are in the context of someone who does not work predominantly in mental health, but does work on cohort studies and recognises that for those studies we work on we often forget how much is ‘inside our heads’ and may be assumed to much of readers who are nothing like as familiar as ‘we are’ (in this case the authors of this paper who know GS-SFHS and STRADL in considerable detail). There were places where I felt a bit lost and could have done with more detail that publication on WOR supports.

 Please note I felt obliged to tick 'not approved' as that was the only option that states I am suggesting revisions that I feel have to be undertaken. I think this is an important paper that should be indexed and widely read. I do think that some of my key concerns to require that changes are made to the paper.

 Specifically:

1. Some brief description of the selection criteria for GS-SFHS and its initial aims would be really useful. Did it cover the whole of Scotland? Was it family/house based inclusion criteria? What were the initial inclusion and exclusion criteria? How many in that cohort as a whole have been lost to follow-up? A paragraph providing this background context (rather that relying on the reader knowing GS-SFHS or going and reading that cohort profile) would be really useful.
2. Similarly, some detail on STRADL – i.e. the first (remote) assessment I think is essential – what were the eligibility criteria at the start of STRADL? What was the response to that first remote STRADL assessment? What data were collected in that remote STRADL? And were the aims of that remote STRADL assessment the same as those for this more detailed face-to-face follow-up? This to me is essential both to understand the value of this recent face-to-face assessment and to understand some of the demographic differences presented and the potential for selection bias in STRADL as a whole and also this face-to-face assessment
3. Related to point 2 above I think that Figure 1 needs to be amended so that STRADL remote is also included – this way a clearer picture of the relation between that and the current face-to-face is clear.
4. The overall response of the 5649 invited to take part in the face-to-face presented here is 21% (N = 1188) that fact is never mentioned and it really has to be – it should be stated in the abstract; clearly shown in Figure 1 and stated in the text related to that figure and needs to be discussed in more detail in the discussion limitations (again it is not mentioned as a limitation in that section).
5. It is stated that STRADL recruitment and data collection ended in May 2019 but I could not find any statement showing when it started. As this is a study about longitudinal analyses that to me seems essential to report. As noted below, I found the chronology and what was truly available for repeat longitudinal assessment (i.e. of change in an exposure or an outcome) or for prospective analyse (i.e. of an exposure at one time point with an outcome at a later time point) confusing. I think a figure that showed the chronology on a time scale of the baseline GS-SFHS (2006-2011), STRADL-remote (initial formation of STRADL; 2015? month of start and finish) and STRADL face-to-face (?? To May 2019) – on a horizontal date metre with an indication of median and full range of time between these – would be really useful. It would also be useful to include in that for the Walker and the ACONF the baseline collection of data in those (e.g. could be an arrow back to 1956 for ACONF) and also the date ranges of different linkages (I think for some hospital records may be only back to 90s and may differ between ‘psychiatric’ diagnoses and ‘physical’ diagnoses).
6. I found the content, including column headings of Table 1 confusing, in particular as the content often seemed to be contradicted by the text that referred to that table.
  Specifically:
  1. The chronology and repeats seemed erroneous. How can the GS-SFHS be a repeat when that is the baseline measure .. yet the heading calls it ‘repeat’. Similarly, the STRADL remote is repeat but that was also before the current face-to-face. As above no specific date is given for STRADL face-to-face it simply says ‘current’, which isn’t quite true if recruitment and data collection finished in May 2019 – what is needed is a start and end date for each of these column headings, and I would order chronologically and take out ‘repeat’ from any of the column headings as it is clear if something has been measured more than once from the entries (or at least should be).
  2. In the text it states that cognitive function measures assessed at STRADL face-to-face are the same as those done at GS-SFHS in several places (including in the discussion as a strength that these were “deliberately the same”), but in Table 1 under cognitive measurements there is no overlap in the ticks for measures from GS-SFHS and STRADL face-to-face. If some were deliberately repeated there should be a tick for both GS-SFHS and STRADL face-to-face.
  3. The text also implies that DNA was taken again at the face-to-face but there is only a tick for that at GS-SFHS & the same applies for some of the plasma biomarkers.
7. Could the authors clarify when the demographic data presented in Table 4 were taken from. I assume that they were all taken from GS-SFHS baseline assessment, which would be the appropriate comparison (i.e. suggesting that those who agree to participate in the initial STRADL study (the remote one) were older at that baseline than those who did not respond/take part in that and those who took part in STRADL face-to-face). Wherever they are taken from it is important this is stated in the text describing the table and title / footnote to the table; at the moment I could not see it anywhere.
8. It would be useful in Table 4 and/or in text to have some indication of demographic differences in the two birth cohorts (Walker and ACONF) at each of the assessments. This seems important to me as the older age in STRADL face-to-face (and possibly STRADL remote but not possible to tell without further information on that) could be due to oversampling form those birth cohorts. The Walker cohort were born 1952 to 1966 and the ACONF in 1956 – if that makes them generally older that the GS-SFHS to me that has different implications for selection than if they are similarly aged to the rest of GS-SFHS.
9. For a cohort profile – which in my book is about telling the international academic community what is available and what it might be used for and where there are limitations and caution required – I was very surprised at the lack of any discussion of limitation. To me the following all need detailed discussion:
  1. Sample size. The aim of STRADL and in particular the face-to-face follow-up described here is said to be to “ *examine the interaction between genetic and environmental factors that increase risk and occurrence of different MDD subtypes, and assess common and distinct mechanisms and clinical trajectories of MDD phenotypes. Additionally, STRADL aims to assess individual resilience, or the ability to adapt positively and ‘avoid’ psychopathology despite exposure to known risk factors such as stress, early-life adversity, and family history*” ‘Interactions’ and ‘subtypes’ (i.e. with the two together subgroups within subgroups) require very large sample sizes to give meaningful results and it is not clear to me how this will be possible with just over 1000 participants. Some examples of clinically meaningful results for stratified medicine / precision prevention (the focus of the introduction and justification for the STRADL sub study) would be really valuable.
  2. Selection bias As noted previously the 21% of those eligible and invited to the STRADL face-to-face study that is the focus of this profile seems to be ignored. There have been a very large number of publications recently that have described issues related to this -including when exploring genetic associations (and particularly in relation to gene-environment interactions) and a clear discussion of that in relation to this study is necessary.
  3. Availability of data for the aims Related to both points above it is unclear from what is written how much missing data will impact on the research that this study aims to particularly address. For example, of the 1188 recruited 544 (~47%) will have prospectively collected birth / early life data (i.e. those in the Walker and ACONF) birth cohorts) but the remaining 53% will not. And whilst Table 2 & 3 provide some reassurance they do not cover the early life data and even with small amounts of missing data (as low as 2-3%) for single variables once one tries to combine these the numbers with complete data for all models/analyses can be considerably smaller than 100%. Related to this could the authors clarify where data in Tables 2 & 3 come from. Are they from one particular assessment (of the three that the 1188 are likely to have participated in) or from any – e.g. could weight and height be available on 99.9% because they are coded ‘available’ if they had at least one measure from any of the three assessments). Again this information is essential to understanding the potential for repeat measures and prospective analyses that the study aims to address.
  4. Relevance of data for contemporary populations I appreciate that all of our studies are somewhat restricted in terms of their target populations, but as this study (based on ages of different groups) were born in the 1950s and 60s predominantly some discussion of how the data collected for early life exposures will have been influenced by norms and cultures at the time, including how people now in their 50s and 60s might remember or be willing to retrospectively report what would now-adays be considered ‘adversity’ but may not have been at the time. How will possible residual confounding be dealt with for analyses of the early life exposures when these may not have been collected. For example, smoking during pregnancy was not recorded in ACONF)

As well as discussing these limitations I think one actual analysis for this paper relevant to the aims of STRADL would be valuable to give the authors and readers a sense of what is truly possible. For example, one could try to explore whether there is an interaction between childhood adversity and adult alcohol consumption and one of the facel-to-face MRI or cognitive function continuously measured outcomes. That would provide some evidence of power in relation to precision of any effect estimates an idea of missing data when trying to ensure all potential confounders are accounted for and would mean having to discuss any results in the context of possible selection bias.

 Minor comments

- The following sentences in the abstract “Some of the questionnaire and cognitive data were first assessed at the GS:SFHS baseline assessment between 2006-2011 **, thus** providing longitudinal measures of depression and resilience. **Similarly,** routine NHSdata and early-life variables are linked to STRADL data, further providing opportunities for longitudinal analysis” (my highlight) read a little oddly to me. As the date for the STRADL study describe here is not prevented it is not clear how / why these provide longitudinal measures. Also it feels like depression and resilience are being used as interchangeable with cognitive function to some extent in the first of these two sentences.
- In the introduction “Thus, it is important for treatment to shift from the current “trial and error” approach, towards **personalised ** and preventative forms of treatment **for individuals ** with markedly different disease mechanisms.” As we cannot make inference to individuals or personalise (i.e. to an individual) treatments from cohort or RCT studies I would change this to the terms that are more widely accepted – ‘stratified medicine’ and ‘precision prevention’
- It is worth clarifying (for a wide international audience) that Aberdeen is in Grampian (as you do for Dundee and Tayside).
- Under the summary of biological samples, you mention repeat genetic analyses. Might be worth changing to repeat DNA Methylation as genetic variants will not change over time. Also I would clarify in that section when you refer to ‘epigenomic status’ you mean DNA methylation and transcription (you refer to RNA later). Also, worth clarifying that DNA methylation will be in white blood cells only (I think that is correct – if not then state witch other tissues). From the text I understood that DNA would be extracted from samples again a the STRADLE face-to-face but there is not tick in Table 1 to confirm that?
- In Table 1 it appears as if on all participants DNA from blood and saliva are available whereas that is not the case. I would suggest where you first mention extracting DNA from saliva when blood cells are not available you give the N & % in parentheses where that had to be done & in Table 1 just have one line ‘Extracted DNA’ and a footnote stating that for 97.5% that was from white blood cells with the remaining 2.5% being from saliva

Is the work clearly and accurately presented and does it cite the current literature?

Partly

If applicable, is the statistical analysis and its interpretation appropriate?

Not applicable

Are all the source data underlying the results available to ensure full reproducibility?

Yes

Is the study design appropriate and is the work technically sound?

Partly

Are the conclusions drawn adequately supported by the results?

Partly

Are sufficient details of methods and analysis provided to allow replication by others?

Partly

Reviewer Expertise:

Epidemiology

I confirm that I have read this submission and believe that I have an appropriate level of expertise to state that I do not consider it to be of an acceptable scientific standard, for reasons outlined above.

# Response

**Published:** 2021-06-08

**Authors:** Tina Habota

**Affiliations:** University of Aberdeen, UK

- Some brief description of the selection criteria for GS-SFHS and its initial aims would be really useful. Did it cover the whole of Scotland? Was it family/house-based inclusion criteria? What were the initial inclusion and exclusion criteria? How many in that cohort as a whole have been lost to follow-up? A paragraph providing this background context (rather than relying on the reader knowing GS-SFHS or going and reading that cohort profile) would be really useful.

*Response:*

 The premise and aims of GS:SFHS were described briefly, but we have included further details that describes the selection process/criteria addressing the reviewer’s comment:

*“GS:SFHS included participants aged 35-65 years who were identified at random from collaborating medical practices across Scotland, with some family members further afield. Initially, only Glasgow and Tayside areas were involved, but the study was extended in 2010 to include Ayrshire, Arran and Northeast Scotland, with the age range also broadened (to 18-65 years). Participants were included if: they met the age criteria; had capacity to give informed consent; and could identify at least one first-degree relative who would also participate. Follow-up of participants was done through the NHS Scotland Community Health Index (CHI): 7% of the original cohort could not be matched; no participants withdrew; and ~1,200 had died”.*

- Similarly, some detail on STRADL – i.e. the first (remote) assessment I think is essential – what were the eligibility criteria at the start of STRADL? What was the response to that first remote STRADL assessment? What data were collected in that remote STRADL? And were the aims of that remote STRADL assessment the same as those for this more detailed face-to-face follow-up? This to me is essential both to understand the value of this recent face-to-face assessment and to understand some of the demographic differences presented and the potential for selection bias in STRADL as a whole and also this face-to-face assessment

*Response:*

 Thank you for this comment and we agree that this could be clarified further in the manuscript. We have clarified the differences between the two STRADL waves by elaborating and clarifying the aims of STRADL remote follow-up:

*“* *The first wave of STRADL included depression-focused follow-up assessment of GS:SFHS, which involved remote questionnaires that specifically assessed aspects of psychological resilience, coping style, and response to psychological distress; study protocol and cohort characteristics are described elsewhere [12]”.*

 We have also included a brief description of STRADL remote study:

*“Those who participated in GS:SFHS were invited to take part in the STRADL remote follow-up based on the following eligibility criteria if: they had given consent for re-contact; were living in Scotland; and had a CHI number. 9,618 GS:SFHS responded to the invitation, including 2,460 unrelated individuals and 2,460 families (7,158 individuals) of between 2 and 18 family members”.*

- Related to point 2 above I think that Figure 1 needs to be amended so that STRADL remote is also included – this way a clearer picture of the relation between that and the current face-to-face is clear.

*Response:*

 We agree with the reviewers and have added this additional information to Figure.

- The overall response of the 5649 invited to take part in the face-to-face presented here is 21% (N = 1188) that fact is never mentioned and it really has to be – it should be stated in the abstract; clearly shown in Figure 1 and stated in the text related to that figure and needs to be discussed in more detail in the discussion limitations (again it is not mentioned as a limitation in that section).

*Response:*

 We agree that this was not sufficiently clear in our original submission and we thank the reviewer for drawing our attention to this issue.  We have now clarified the participation rate which states:

*“….* *we consented and tested 1,188 (72.2%) of positive respondents across Aberdeen (n = 582) and Dundee (n = 606) sites. This meant that we tested 21% of the n = 5,649 who were initially invited to participate”*.

 We did not include this statistic in Figure 1 because we only report sample sizes and not proportions, but the sample size is consistent with the quoted proportion.

- It is stated that STRADL recruitment and data collection ended in May 2019 but I could not find any statement showing when it started. As this is a study about longitudinal analyses that to me seems essential to report. As noted below, I found the chronology and what was truly available for repeat longitudinal assessment (i.e. of change in an exposure or an outcome) or for prospective analyse (i.e. of an exposure at one time point with an outcome at a later time point) confusing. I think a figure that showed the chronology on a time scale of the baseline GS-SFHS (2006-2011), STRADL-remote (initial formation of STRADL; 2015? month of start and finish) and STRADL face-to-face (?? To May 2019) – on a horizontal date metre with an indication of median and full range of time between these – would be really useful. It would also be useful to include in that for the Walker and the ACONF the baseline collection of data in those (e.g. could be an arrow back to 1956 for ACONF) and also the date ranges of different linkages (I think for some hospital records may be only back to 90s and may differ between ‘psychiatric’ diagnoses and ‘physical’ diagnoses).

*Response:*

 Thank you for your comments above. We have now included an additional table that shows all existing data sets that are linked to the STRADL face-to-face study. We have now introduce Table 1 and state: *“* *Table 1 shows the linkage between the current study and existing datasets”. *

- I found the content, including column headings of Table 1 confusing, in particular as the content often seemed to be contradicted by the text that referred to that table. Specifically: 
- The chronology and repeats seemed erroneous. How can the GS-SFHS be a repeat when that is the baseline measure .. yet the heading calls it ‘repeat’. Similarly, the STRADL remote is repeat but that was also before the current face-to-face. As above no specific date is given for STRADL face-to-face it simply says ‘current’, which isn’t quite true if recruitment and data collection finished in May 2019 – what is needed is a start and end date for each of these column headings, and I would order chronologically and take out ‘repeat’ from any of the column headings as it is clear if something has been measured more than once from the entries (or at least should be).

*Response:*

 Table 2 (previously Table 1) lists all of the variables collected in STRADL face to face assessment compared to STRADL remote follow-up and GS, and specifically we have indicated which variables are “new” (column 1). Then we have also indicated which were “repeated” from STRADL remote study and GS baseline assessment (columns 2 and 3).

- In the text it states that cognitive function measures assessed at STRADL face-to-face are the same as those done at GS-SFHS in several places (including in the discussion as a strength that these were “deliberately the same”), but in Table 1 under cognitive measurements there is no overlap in the ticks for measures from GS-SFHS and STRADL face-to-face. If some were deliberately repeated there should be a tick for both GS-SFHS and STRADL face-to-face.

*Response:*

 Table 2 (previously Table 1) lists all of the variables collected in STRADL face to face assessment, which included eight cognitive tasks. Four cognitive tests (Logical Memory; Digit Symbol Coding; Verbal Fluency; Mill Hill Vocabulary) were repeated from GS baseline assessment; and four (Matrix Reasoning; Bristol Emotion Recognition test; Affective Go/No-Go task; Cambridge Gambling task) were new tests only administered in face-to-face assessment.

- The text also implies that DNA was taken again at the face-to-face but there is only a tick for that at GS-SFHS & the same applies for some of the plasma biomarkers.

*Response:*

 DNA was obtained from whole blood at the STRADL face-to-face assessment. Table 2 shows all of the variables that were a part of the STRADL face to face assessments – therefore we have now clearly indicated that this is a repeat from the GS baseline assessment. Table 4 shows the proportion of DNA obtained via blood sample and saliva sampling. 

- Could the authors clarify when the demographic data presented in Table 4 were taken from. I assume that they were all taken from GS-SFHS baseline assessment, which would be the appropriate comparison (i.e. suggesting that those who agree to participate in the initial STRADL study (the remote one) were older at that baseline than those who did not respond/take part in that and those who took part in STRADL face-to-face). Wherever they are taken from it is important this is stated in the text describing the table and title / footnote to the table; at the moment I could not see it anywhere.

*Response:*

 The demographic data from Table 6 (perviously Table 5) was taken at time of assessment at each of the study points (GS, STRADL remote follow-up, and STRADL face-to-face assessment). We have noted this in a footnote to the table on page 23:

*“* *Data were extracted from time of each assessment point”.  *

- It would be useful in Table 4 and/or in text to have some indication of demographic differences in the two birth cohorts (Walker and ACONF) at each of the assessments. This seems important to me as the older age in STRADL face-to-face (and possibly STRADL remote but not possible to tell without further information on that) could be due to oversampling form those birth cohorts. The Walker cohort were born 1952 to 1966 and the ACONF in 1956 – if that makes them generally older that the GS-SFHS to me that has different implications for selection than if they are similarly aged to the rest of GS:SFHS.

*Response:*

 Under the section “what are the key findings”, we have elaborated on the age differences between groups at STRADL face-to-face assessment, as below:

*“Analysis of group differences for age showed that participants who are part of ACONF are generally older (M = 62.32; SD = 1.55) compared to participants in the Walker cohort and wider GS:SFHS participants (ts* *³* *6.28; ps < .001). However, age did not differ (t = 1.83; p = .068) between participants in the Walker cohort (M = 59.48; SD = 6.76) and those in GS:SFHS (M = 58.28; SD = 12.63)”.  *

- For a cohort profile – which in my book is about telling the international academic community what is available and what it might be used for and where there are limitations and caution required – I was very surprised at the lack of any discussion of limitation. To me the following all need detailed discussion:
- Sample size. The aim of STRADL and in particular the face-to-face follow-up described here is said to be to “ *examine the interaction between genetic and environmental factors that increase risk and occurrence of different MDD subtypes, and assess common and distinct mechanisms and clinical trajectories of MDD phenotypes. Additionally, STRADL aims to assess individual resilience, or the ability to adapt positively and ‘avoid’ psychopathology despite exposure to known risk factors such as stress, early-life adversity, and family history*” ‘Interactions’ and ‘subtypes’ (i.e. with the two together subgroups within subgroups) require very large sample sizes to give meaningful results and it is not clear to me how this will be possible with just over 1000 participants. Some examples of clinically meaningful results for stratified medicine / precision prevention (the focus of the introduction and justification for the STRADL sub study) would be really valuable.

*Response:*

 No sample size calculation was provided for the recruiting participants in the current investigation. A larger sample of individuals was originally planned, which was reduced in light of UK Biobank’s stated plans to recruit upwards of 10,000 participants for neuroimaging. The study plan was changed to reduce the number of participants included for imaging purposes – but representing at least a 4-fold increase on the current average sample size of a depression imaging cohort study. We estimated that this sample would have sufficient power to detect previously reported true differences from studies of approximately 50 cases and 50 controls, and detect differences explaining approximately 50% of the variance previously reported by earlier studies. However, this power calculation was not conducted prospectively and we have not therefore included this in the text of the manuscript, although we agree that this is a limitation.

- Selection bias As noted previously the 21% of those eligible and invited to the STRADL face-to-face study that is the focus of this profile seems to be ignored. There have been a very large number of publications recently that have described issues related to this -including when exploring genetic associations (and particularly in relation to gene-environment interactions) and a clear discussion of that in relation to this study is necessary.

*Response:*

 Under the “Strengths and Limitations” section, we have elaborated on the above point and state:

*“A l* *imitation of this study includes possible selection bias as we tested only 21% of the total pool of eligible and invited participants (n = 5,649). As with many longitudinal population studies, participants in this cohort were more likely to be of good health, and come from more advantaged backgrounds such as higher education and better socioeconomic circumstances than the population in general – findings that are similar to GS:SFHS and STRADL remote follow-up cohort profiles [8, 12], and UK Biobank [17]. Further,* *because our cohort was of good psychological health at the time of testing, it seems possible that we did not capture as many people with poorer psychological health as what might be available in the wider cohort”. * 

- Availability of data for the aims Related to both points above it is unclear from what is written how much missing data will impact on the research that this study aims to particularly address. For example, of the 1188 recruited 544 (~47%) will have prospectively collected birth / early life data (i.e. those in the Walker and ACONF) birth cohorts) but the remaining 53% will not. And whilst Table 2 & 3 provide some reassurance they do not cover the early life data and even with small amounts of missing data (as low as 2-3%) for single variables once one tries to combine these the numbers with complete data for all models/analyses can be considerably smaller than 100%. Related to this could the authors clarify where data in Tables 2 & 3 come from. Are they from one particular assessment (of the three that the 1188 are likely to have participated in) or from any – e.g. could weight and height be available on 99.9% because they are coded ‘available’ if they had at least one measure from any of the three assessments). Again this information is essential to understanding the potential for repeat measures and prospective analyses that the study aims to address.

*Response:*

 Linkage to early-life variables will be carried out through data available from the ACONF and Walker birth cohorts, but also through routine NHS data. Whilst we have provided the proportion of available data in Tables 3 and 4 for the current study, we do not have the missing values for the NHS datasets as these are continuously updated and missing values differ greatly on an item-by-item basis. The impact of any missing data will, however, need to be addressed in individual analyses and papers.

- Relevance of data for contemporary populations I appreciate that all of our studies are somewhat restricted in terms of their target populations, but as this study (based on ages of different groups) were born in the 1950s and 60s predominantly some discussion of how the data collected for early life exposures will have been influenced by norms and cultures at the time, including how people now in their 50s and 60s might remember or be willing to retrospectively report what would now-adays be considered ‘adversity’ but may not have been at the time. How will possible residual confounding be dealt with for analyses of the early life exposures when these may not have been collected. For example, smoking during pregnancy was not recorded in ACONF).

*Response:*

 Thank you for your comments above, with which we agree – particularly on the issue of retrospective reporting of adversity. We have included a discussion under "Strengths and Limitations" that addresses this point:

*“As the present study is based on ages of different cohorts including people born in the 1950s onwards, early life variables are likely to have been influenced by cultural and societal norms of that time. For example, people born in the 1950s and 1960s may not have considered housing circumstances such as crowdedness as an ‘adverse experience’, contrary to more contemporary perspectives. These possible confounding variables will be considered in downstream analyses, but it may be difficult to completely mitigate their effects”.*

- As well as discussing these limitations I think one actual analysis for this paper relevant to the aims of STRADL would be valuable to give the authors and readers a sense of what is truly possible. For example, one could try to explore whether there is an interaction between childhood adversity and adult alcohol consumption and one of the face-to-face MRI or cognitive function continuously measured outcomes. That would provide some evidence of power in relation to precision of any effect estimates an idea of missing data when trying to ensure all potential confounders are accounted for and would mean having to discuss any results in the context of possible selection bias.

*Response:*

 We agree that this would be an interesting analysis, but we would not wish to add it to this paper. Our aims for this paper were to focus on describing the baseline cohort results without analyses that address specific study aims – these are currently being undertaken in a series of separate analyses and papers. The additional methods required to address this suggestion would also complicate and extend a paper that is already fairly complex.

 Minor comments

- The following sentences in the abstract “Some of the questionnaire and cognitive data were first assessed at the GS:SFHS baseline assessment between 2006-2011 **, thus** providing longitudinal measures of depression and resilience. **Similarly,** routine NHS data and early-life variables are linked to STRADL data, further providing opportunities for longitudinal analysis” (my highlight) read a little oddly to me. As the date for the STRADL study describe here is not presented it is not clear how / why these provide longitudinal measures. Also it feels like depression and resilience are being used as interchangeable with cognitive function to some extent in the first of these two sentences.

*Response:*

 Thank you for raising these points, which we now clarify in the Abstract:

*“* *Some of the questionnaire and cognitive data were first assessed at the GS:SFHS baseline assessment between 2006-2011, thus providing longitudinal measures relevant to the study of depression, psychological resilience, and cognition. In addition, routinely collected historic NHS data and early-life variables are linked to STRADL data, further providing opportunities for longitudinal analysis. Recruitment has been completed and we consented and tested 1,188 participants.”*

  

- In the introduction “Thus, it is important for treatment to shift from the current “trial and error” approach, towards **personalised ** and preventative forms of treatment **for individuals ** with markedly different disease mechanisms.” As we cannot make inference to individuals or personalise (i.e. to an individual) treatments from cohort or RCT studies I would change this to the terms that are more widely accepted – ‘stratified medicine’ and ‘precision prevention’

*Response:*

 We agree and have changed the text as follows:

*“Thus, it is important for treatment to shift from the current “trial and error” approach, towards precision prevention and stratified medicine based on markedly different disease mechanisms”.*

- It is worth clarifying (for a wide international audience) that Aberdeen is in Grampian (as you do for Dundee and Tayside).

*Response:*

 We have now clarified this in the manuscript.

- Under the summary of biological samples, you mention repeat genetic analyses. Might be worth changing to repeat DNA Methylation as genetic variants will not change over time. Also I would clarify in that section when you refer to ‘epigenomic status’ you mean DNA rillmethylation and transcription (you refer to RNA later). Also, worth clarifying that DNA methylation will be in white blood cells only (I think that is correct – if not then state which other tissues). From the text I understood that DNA would be extracted from samples again at the STRADLE face-to-face but there is not tick in Table 1 to confirm that?

*Response:*

 Thank you for these comments. We have clarified this point and state:

*“We collected laboratory blood samples for genetic and additional genomic analyses, including the study of DNA methylation, transcription (RNA) and protein expression.”*

 DNA samples were first collected in GS baseline assessment thus Table 2 (previously Table 1) has a tick to indicate this, and there is no tick under the STRADL face to face assessment because it is not a “new” variable.

- In Table 1 it appears as if on all participants DNA from blood and saliva are available whereas that is not the case. I would suggest where you first mention extracting DNA from saliva when blood cells are not available you give the N & % in parentheses where that had to be done & in Table 1 just have one line ‘Extracted DNA’ and a footnote stating that for 97.5% that was from white blood cells with the remaining 2.5% being from saliva

*Response:*

 Thank you for this comment. Table 2 (previously Table 1) shows all of the variables that were collected overall in the study, whereas Table 4 (previously Table 3) shows the specific proportion of available date – we have amended Table 4 so that it states the total proportion of DNA available (99.2), but we include a footnote that states: 

 “96.5% of DNA was extracted via blood sample and 2.7% via saliva sampling.”
